# Defining metabolic flexibility in hair follicle stem cell induced squamous cell carcinoma

**DOI:** 10.1126/sciadv.adn2806

**Published:** 2024-09-20

**Authors:** Carlos Galvan, Aimee A. Flores, Victoria Cerrilos, Itzetl Avila, Conor Murphy, Wilson Zheng, Heather R. Christofk, William E. Lowry

**Affiliations:** ^1^ Department of Molecular Cell and Developmental Biology, UCLA, Los Angeles, CA 90095, USA.; ^2^ Molecular Biology Institute, UCLA, Los Angeles, CA 90095, USA.; ^3^ Broad Stem Cell Research Center, UCLA, Los Angeles, CA 90095, USA.; ^4^ Department of Biological Chemistry, DGSOM, UCLA, Los Angeles, CA 90095, USA.; ^5^ Jonsson Comprehensive Cancer Center, UCLA, Los Angeles, CA 90095, USA.; ^6^ Department of Medicine, DGSOM, UCLA, Los Angeles, CA 90095, USA.

## Abstract

We previously showed that inhibition of glycolysis in cutaneous squamous cell carcinoma (SCC)–initiating cells had no effect on tumorigenesis, despite the perceived requirement of the Warburg effect, which was thought to drive carcinogenesis. Instead, these SCCs were metabolically flexible and sustained growth through glutaminolysis, another metabolic process frequently implicated to fuel tumorigenesis in various cancers. Here, we focused on glutaminolysis and genetically blocked this process through glutaminase (GLS) deletion in SCC cells of origin. Genetic deletion of GLS had little effect on tumorigenesis due to the up-regulated lactate consumption and utilization for the TCA cycle, providing further evidence of metabolic flexibility. We went on to show that posttranscriptional regulation of nutrient transporters appears to mediate metabolic flexibility in this SCC model. To define the limits of this flexibility, we genetically blocked both glycolysis and glutaminolysis simultaneously and found the abrogation of both of these carbon utilization pathways was enough to prevent both papilloma and frank carcinoma.

## Teaser

In this study, the limits of metabolic flexibility in squamous cell carcinoma are defined and mediated by nutrient transporters.

## INTRODUCTION

Cutaneous squamous cell carcinoma (SCC) is known to initiate in the epidermis due to an accumulation of mutations in genes such as *Ras*, *Notch*, *P53*, etc. (*1*–*4*). We and others showed that these cancers can arise due to transformation of hair follicle stem cells (HFSCs) using murine transgenic and chemically induced carcinogenesis protocols (*5*, *6*). In humans, these cancers are typically treated through surgical resection but, if left untreated, can metastasize and be lethal. SCC can also arise in other areas that are much more difficult to treat such as in head and neck SCC, leading to much higher rates of mortality (*2*, *7*–*9*).

SCC and essentially every other solid tumor are known to show evidence of a metabolic transition known as the Warburg effect where cancer cells choose to increase glucose uptake as a nutrient and use it to produce lactate leading to acidification of tissue, a process also known as aerobic glycolysis (*10*, *11*). This observation has led to serious effort to block various targets in the glucose utilization pathway for the treatment of cancer. However, to date, these approaches have not been successful (*12*). We previously showed that genetic deletion of lactate dehydrogenase A (LDHA) in cancer initiating cells of the epidermis led to a marked decrease in the Warburg effect as measured by glucose uptake and lactate production (*13*). However, SCC formed despite lack of LDHA, suggesting that cancer cells do not necessarily rely on glucose metabolism for their growth and transformation. Instead, these data raised the possibility that cancer cells show metabolic flexibility which allows them to grow by up-regulating alternative pathways that generate adenosine triphosphate (ATP) and biosynthesis of key materials to allow for increased proliferation. As potential evidence of flexibility, we also showed that tumors lacking LDHA activity exhibited increased glutamine consumption (*13*); however, we did not experimentally investigate in the previous study whether this increased glutamine metabolism enabled tumor growth in the absence of LDHA.

Glutaminolysis has also emerged as a key metabolic pathway in a variety of cancer models (*14*, *15*). Glutamine is imported into cells via plasma membrane glutamine transporters and can be converted to glutamate through the action of glutaminase (GLS) enzymes in the cytoplasm or mitochondria (*16*). Glutamate can be converted to alpha-ketoglutarate which can enter into the tricarboxylic acid (TCA) cycle to power oxidative phosphorylation and production of ATP in the mitochondria. In addition, recent data show that many human tumors consume lactate through monocarboxylate transporters, and convert it into pyruvate by lactate dehydrogenase, and then use that pyruvate anapleurotically in the TCA cycle (*17*). Therefore, cancer cells can acquire carbon-based nutrients to power the TCA cycle either through uptake of glucose, lactate, or glutamine, all of which have been shown to be up-regulated in many human cancers. Despite numerous studies suggesting that glutaminolysis could be a driver of tumorigenesis, this has yet to be tested genetically in vivo in murine cancer models, particularly in SCC. To date, efforts to block glutaminolysis with small-molecule inhibition of GLS activity have not yet led to clinically available therapies for patients despite intense effort (*15*, *18*–*20*).

In the current study, we use a well-established murine model of SCC coupled with genetic deletion of LDHA and GLS to test the limits of cancer metabolic flexibility (*21*, *22*). In this model, genetic manipulation is inducible in HFSCs, which are known to initiate SCC in murine epidermis (*5**,* *6*). Coupled with chemical carcinogenesis, this model allows for the deletion of metabolic activity just before induction of oncogenesis in adult mice. Because the tumorigenesis begins at the skin surface, the entire process is tractable over time allowing for detailed quantification of oncogenesis and precise measurement of the role various metabolic pathways play in this process. We exploited this model to probe the role of glutaminolysis in SCC initiation or progression and, in doing so, define metabolic flexibility in SCC as well as the limits of that flexibility.

## RESULTS

**Fig. 1.**
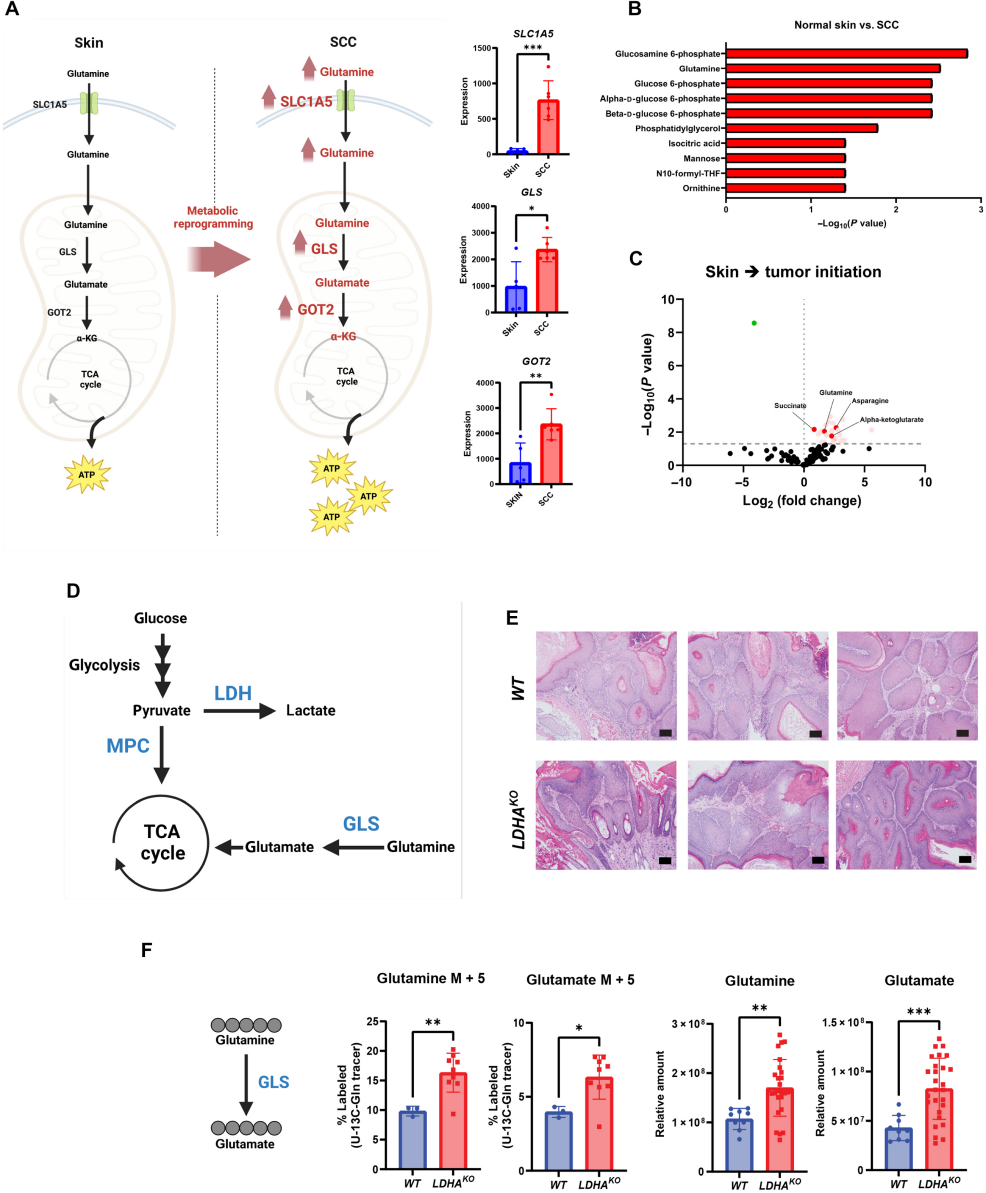
Glutamine metabolism is up-regulated in SCC. (A) Schematic showing metabolic reprogramming in glutamine metabolism from normal skin to SCC. Glutamine is metabolized into TCA cycle, and ATP is synthesized from TCA cycle–derived glutamine. ASCT2 (SLC1A5), GLS, and GOT2 gene expression in skin (*n* = 5) and SCC (*n* = 6). Statistical significance (*P < 0.05, **P < 0.01, and ***P < 0.001) was calculated using a two-tailed *t* test. (**B**) Ontological analysis from metabolomics workbench for metabolic substrates increased in SCC compared to normal skin. THF, tetrahydrofolate. (**C**) Metabolomic pool volcano plot of normal skin versus papilloma (benign tumor) initiated by DMBA/TPA skin chemical carcinogenesis. Dashed lines indicate adjusted *P* ≤ 0.05 or log_2_(fold change) of ≥0 or ≤0. Colored dots represent metabolites significantly increasing (red) or decreasing (green) during the skin to tumor transition. (**D**) Schematic of enzymes used in glycolysis, lactate production, and glutaminolysis. (**E**) Tumors from *WT* and *LDHA^KO^* mice stained for hematoxylin and eosin (H&E). Scale bars, 10 μm. (**F**) Schematic of fully labeled glutamine isotopomer conversion. Data represent percent of M5-labeled glutamine and M5-labeled glutamate in tumors [*n* = 3 (*WT*), *n* = 9 (*LDHA^KO^*)] after ^13^C_5_-glutamine infusion. Metabolic pool data representing relative amounts of glutamine and glutamate in tumors [*n* = 9 (*WT*), *n* = 27 (*LDHA^KO^*)]. Statistical significance (*P < 0.05, **P < 0.01, ***P < 0.001, and ****P < 0.0001) was calculated using a two-tailed *t* test. Figure 1 (A, D, and F) were produced using BioRender.

Glutamine metabolism (Fig. 1A) has previously been implicated as a key metabolic activity in tumor progression in a variety of cancer models (*14**,* *19**,* *20**,* *23*–*25*). Here, we sought to understand whether glutamine metabolism plays a role in tumor initiation or progression of SCC. We previously demonstrated that HFSCs serve as cells of origin for SCC and that the tumors formed share many physiological and metabolic similarities with SCC formed in human skin (*5**,* *26**,* *27*). We acquired transcriptome data from dimethylbenz[*a*]anthracene (DMBA)/12-*O*-tetradecanoylphorbol 13-acetate (TPA)–induced SCC to determine which metabolic pathway genes were altered at the RNA level. We found that many of the genes involved in glutaminolysis and glutamine metabolism elevated in murine SCC derived from HFSCs (Fig. 1A). An ontological analysis for metabolic substrates showed a robust enrichment in gene expression for genes related to mostly glycolysis and glutaminolysis, as expected (Fig. 1B). Ontological analysis for biological processes increased in SCC compared to normal skin demonstrate classical transformations in cancer such as promotion and enhancement of cell division, cell migration, and signaling pathways (fig. S1A), consistent with transcriptome transformation in cancer. We then examined cancer genome data (Gene Expression database of Normal and Tumor tissues, GENT2) to assess the relative mRNA levels in normal versus tumorigenic human tissues, which pointed toward higher expression of GLS, the enzyme that converts glutamine to glutamate as the first step of glutaminolysis, and other enzymes involved in glutamine metabolism in many tumor types (fig. S1, B and C). Next, we performed liquid chromatography mass spectrometry (LCMS)–based metabolomics to measure the relative levels of metabolites in HFSC-induced SCC and found elevated levels of several metabolites involved in glutamine metabolism (Fig. 1C). In addition, in a model of HFSC-induced tumorigenesis, we found that deletion of LDHA did not markedly affect tumor production but did show evidence of metabolic compensation by glutaminolysis (Fig. 1, D to F). On the other hand, transcriptional analysis of LDHA-null tumors did not show changes in expression of genes related to glutaminolysis raising the question of how this compensation was mediated (fig. S1D).

**Fig. 2.**
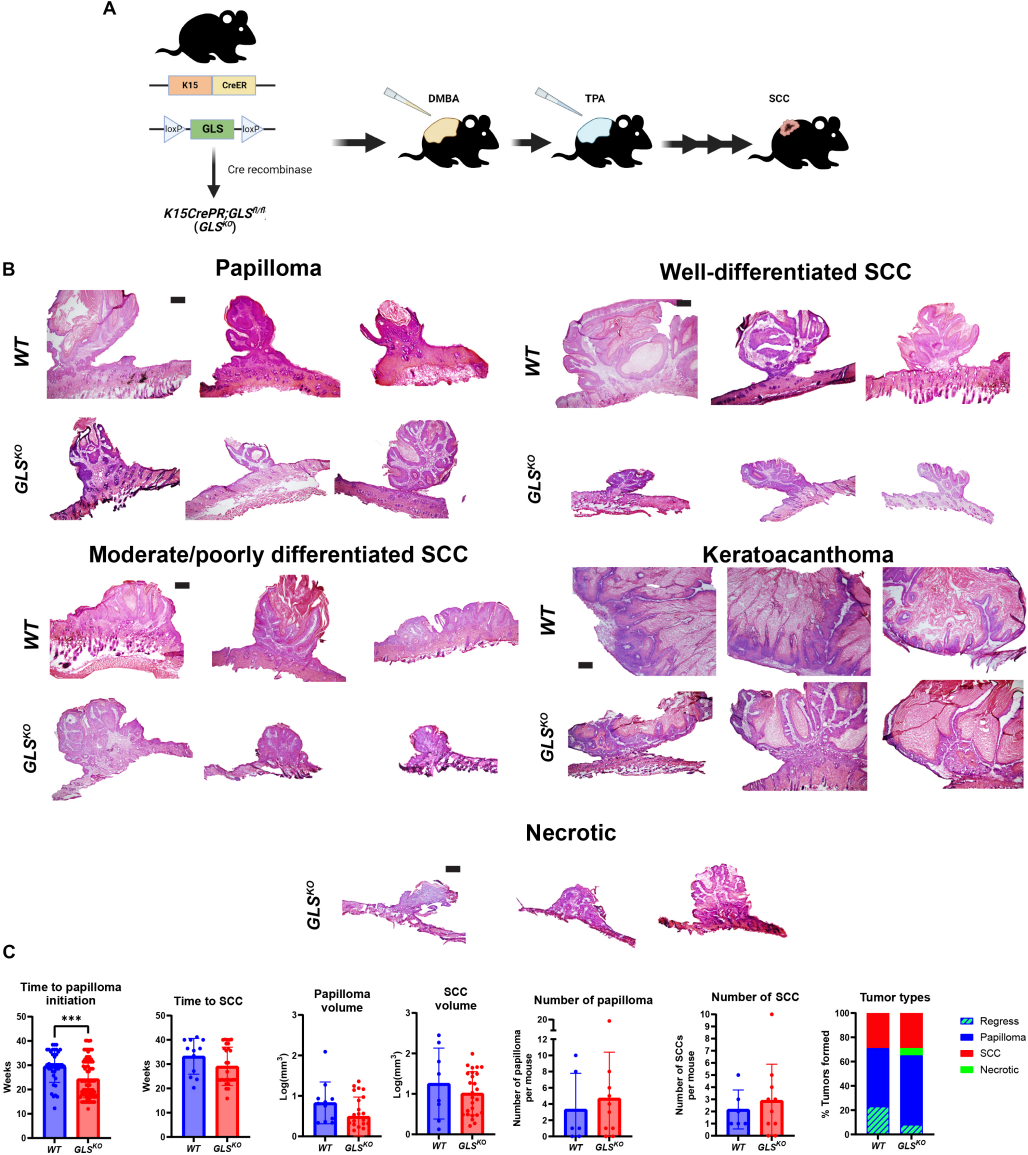
Loss of GLS does not affect SCC initiation, progression, or pathology. (**A**) Schematic of transgenic mice used to knock out GLS in HFSCs coupled with topical SCC chemical carcinogenesis using DMBA and TPA. Figures 2A was produced using BioRender. (**B**) Dorsal tumors from *WT* and *GLS^KO^* mice stained for H&E. Scale bars, 50 μm. (**C**) Quantification of time to papilloma [*n* = 40 (*WT*), *n* = 88 (*GLS^KO^*)] initiation and SCC [*n* = 12 (*WT*), *n* = 29 (*GLS^KO^*)] formation. Each data point represents a tumor of that genotype. Quantification of volume of papilloma [*n* = 11 (*WT*), *n* = 25 (*GLS^KO^*)] and SCC [*n* = 8 (*WT*), *n* = 26 (*GLS^KO^*)]. Each data point represents a tumor of that genotype. Quantification of the number of papilloma [*n* = 6 (*WT*), *n* = 10 (*GLS^KO^*)] and SCC [*n* = 6 (*WT*), *n* = 10 (*GLS^KO^*)] formed per mice. Each data point represents a mouse of that genotype. Data shown represent tumors present at the end of the experiment. Quantification of percent and types of tumors formed per genotype: *WT* (papilloma = 48%; SCC = 30%; regress = 23%; necrotic = 0%) and *GLS^KO^* (papilloma = 57%; SCC = 30%; regress = 8%; necrotic = 6%). Data shown represent tumor quantifications from the beginning to the end of the experiment.

Since SCC tumors showed increased glutaminolysis-related gene expression, we sought to determine the role of glutaminolysis in the initiation or progression of SCC through deletion of GLS in HFSCs before initiating tumorigenesis. Crossing mice floxed for GLS (GLS1 fl/fl, the Jackson Laboratory) with mice transgenic for K15-CrePR allowed for an inducible deletion of GLS in HFSCs upon administration of the progesterone inhibitor mifepristone (Fig. 2A). To induce tumorigenesis, we relied on the established chemical carcinogenesis protocol using DMBA as a mutagen followed by repeated stimulation of proliferation by TPA (*22*). Both *WT* and *GLS^KO^* models produced papilloma, well-differentiated SCC, moderately differentiated SCC, and keratoacanthoma (Fig. 2B). We quantified time to tumor formation, number of tumors, and volume of tumors but did not detect any statistically significant differences in cancer formation in mice with or without GLS expression in cancer cells of origin (Fig. 2C). On the other hand, we did find that 6% of tumors that formed in mice with GLS deletion in cancer cells of origin became necrotic, which we did not observe in animals with GLS activity (Fig. 2C).

**Fig. 3.**
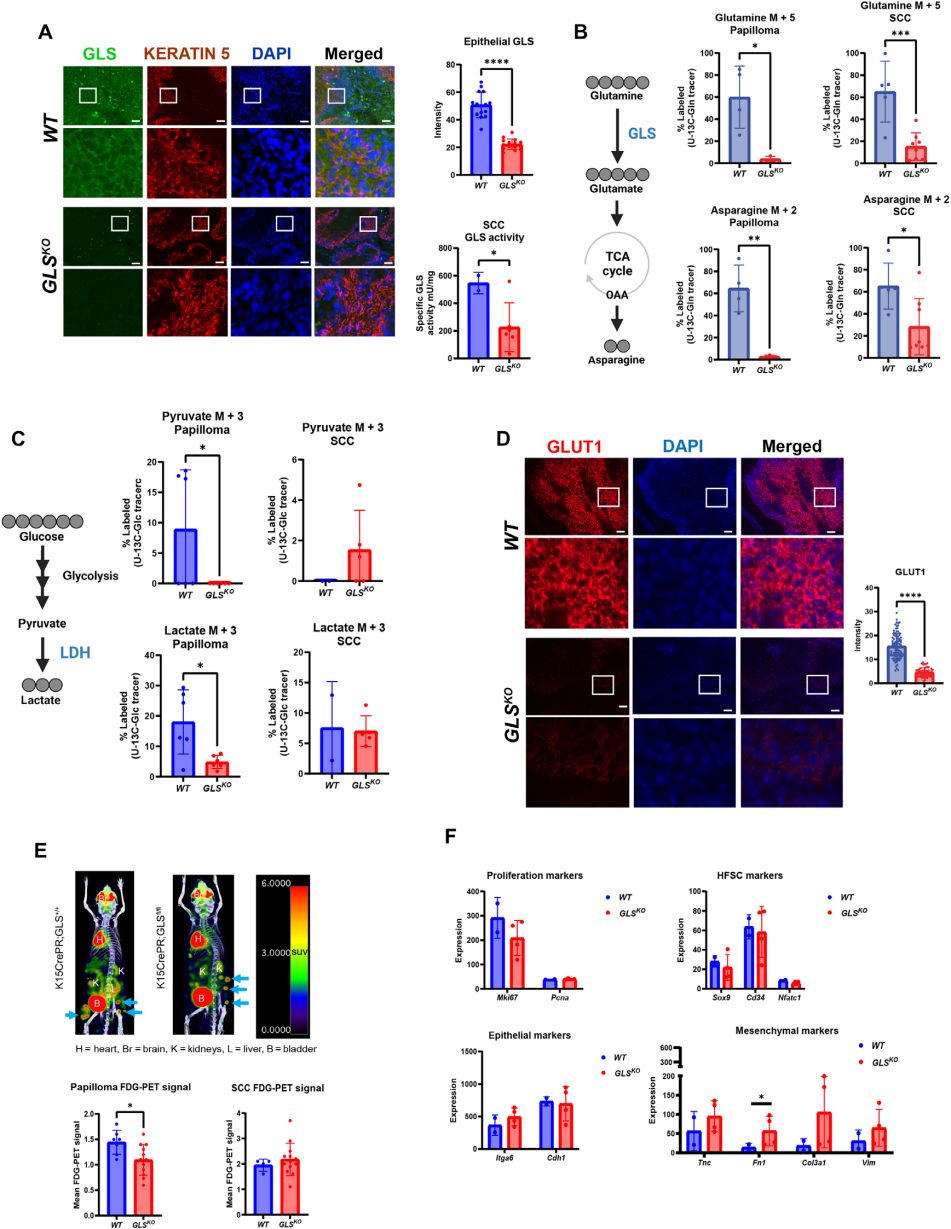
Loss of GLS in tumors alters glutamine and glucose metabolism. (**A**) *WT* or *GLS^KO^* SCC immunostaining for GLS and KERATIN 5, an epidermal marker. Cell nuclei were stained with 4′,6-diamidino-2-phenylindole (DAPI). Quantification of mean intensity epithelial GLS fluorescence in *WT* (*n* = 15) and *GLS^KO^* (*n* = 15) SCCs. GLS activity in *WT* (*n* = 2) and *GLS^KO^* (*n* = 6) SCC lysates. (**B**) Schematic of fully labeled glutamine isotopomer conversion. Data represent percent of M5-labeled glutamine and M2-labeled asparagine in papilloma [*n* = 4 (*WT*), *n* = 3 (*GLS^KO^*)] and SCC [*n* = 5 (*WT*), *n* = 8 (*GLS^KO^*)] after ^13^C_5_-glutamine infusion. OAA, oxaloacetate. (**C**) Schematic of fully labeled glucose isotopomer conversion. Data represent percent of M3-labeled pyruvate and M3-labeled lactate in papilloma [*n* = 6 (*WT*), *n* = 6 (*GLS^KO^*)] and SCC [*n* = 2 (*WT*), *n* = 5 (*GLS^KO^*)] after ^13^C_6_-glucose infusion. (**D**) *WT* or *GLS^KO^* SCC immunostaining for glucose transporter, GLUT1. Cell nuclei were stained with DAPI. Quantification of mean intensity GLUT1 fluorescence in *WT* (*n* = 91) and *GLS^KO^* (*n* = 56) SCCs. Scale bars, 100 μm. (**E**) Mean ^18^F-FDG SUV signal of papilloma [*n* = 7 (*WT*), *n* = 12 (*GLS^KO^*)] and SCC [*n* = 4 (*WT*), *n* = 15 (*GLS^KO^*)]. H, heart; Br, brain; K, kidneys; L, liver; B, bladder. (**F**) RNA-seq data of *WT* (*n* = 2) or *GLS^KO^* (*n* = 5) tumors showing transcription levels of proliferation, HFSC, epithelial, and mesenchymal markers. Figure 3 (B and C) was produced using BioRender. Statistical significance (*P < 0.05, **P < 0.01, ***P < 0.001, and ****P < 0.0001) for (A) to (F) was calculated using a two-tailed *t* test.

To examine whether the genetic deletion of GLS in HFSCs effectively created tumors lacking GLS activity, we used immunostaining, GLS activity assays, and LCMS-based metabolomics. Immunostaining of *WT* tumors showed high GLS expression particularly on the epithelial edge of tumors formed after DMBA/TPA, whereas immunostaining of *GLS^KO^* tumors resulted in negligible GLS staining (Fig. 3A). We also used a GLS activity assay to measure the relative activity of the enzyme in protein lysates generated from *WT* and *GLS^KO^* tumors and found a decrease in GLS activity in the *GLS^KO^* tumors (Fig. 3A). While it is clear that our genetic strategy to eliminate GLS activity from cancer cells of origin and subsequent tumors was successful, we did find cells that were strongly positive for GLS expression in the mesenchyme surrounding the nascent tumors (fig. S2A). These CD45^+^ cells were also positive for CD11b, suggesting that they are macrophages (fig. S2, B and C). Because our genetic strategy was not designed to target immune cells, it is not unexpected to find GLS-positive cells within the mesenchyme, and these will be the subject of future investigation.

To investigate whether GLS-deleted tumors show evidence of metabolic changes despite relative lack of phenotypic change, we performed metabolic tracing with ^13^C-glutamine and LCMS-based metabolomics. Tumors with labeled glutamine showed that both the oxidative and reductive pathways for glutamine utilization were abrogated in GLS-deleted tumors (Fig. 3B), consistent with a loss of GLS activity. On the other hand, metabolomics also showed consistent decreases in glucose conversion to pyruvate and lactate in the absence of GLS in papilloma, but not in SCC (Fig. 3C). Immunostaining for GLUT1, a glucose transporter known to be up-regulated in SCC, showed diminished expression in *GLS^KO^* tumors (Fig. 3D). Furthermore, fluorodeoxyglucose (FDG)–positron emission tomography (PET) imaging, which measures the rate of FDG uptake as a proxy for glucose uptake in tumors of live animals, also showed signs of decreased glucose uptake in the absence of GLS specifically in papilloma (Fig. 3E) (*13*). RNA sequencing (RNA-seq) of *WT* and *GLS^KO^* tumors showed a relatively small number of gene expression differences caused by loss of GLS activity. When looking particularly at genes related to proliferation, epithelial-mesenchymal transition (EMT), or stemness, there were no significant changes (Fig. 3F). Immunostaining for Ki67, proliferation marker, and cleaved caspase 3, apoptosis marker, also showed no changes between *WT* and *GLS^KO^* tumors (fig. S3, A and B). Because the activity of GLS was previously linked to hypoxia signaling, we looked at hypoxia-inducible factor target genes but did not find any differences (fig. S3C). Furthermore, ontological analysis showed that several pathways appeared to be enriched; however, the enrichment was driven by a small number of genes mostly related to ECM and did not point to obvious physiological changes caused by the deletion of GLS (fig. S3D).

**Fig. 4.**
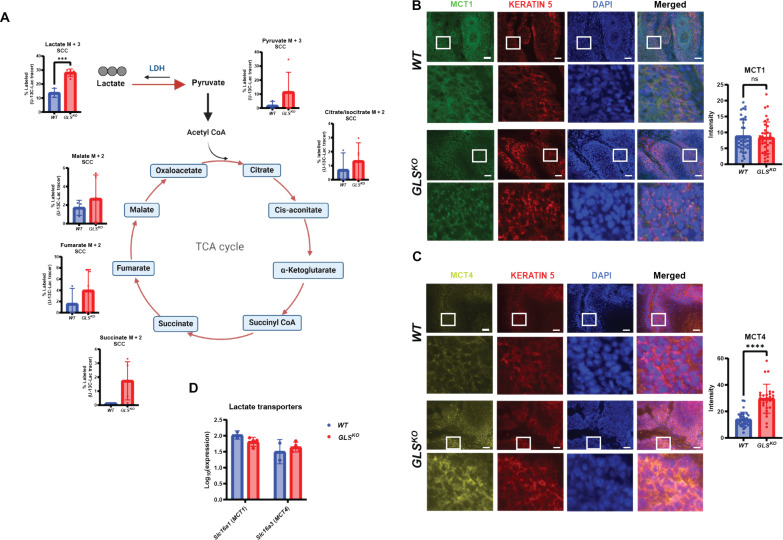
Increased lactate transporter and uptake in *GLS^KO^* SCC. (**A**) Schematic of fully labeled lactate isotopomer conversion. Data represent percent of M3-labeled lactate, M2-labeled citrate/isocitrate, M2-labeled succinate, M2-labeled fumarate, and M2-labeled malate in SCCs [*n* = 3 (*WT*), *n* = 5 (*GLS^KO^*)] after ^13^C_3_-lactate infusion. Statistical significance (***P < 0.001) was calculated using a two-tailed *t* test. CoA, coenzyme A. Figure 4A was produced using BioRender. (**B**) *WT* or *GLS^KO^* SCC immunostaining for lactate transporter, MCT1. Cell nuclei were stained with DAPI. Quantification of mean intensity MCT1 fluorescence in *WT* (*n* = 39) and *GLS^KO^* (*n* = 36) SCCs. ns, not significant. Scale bars, 100 μm. (**C**) *WT* or *GLS^KO^* SCC immunostaining for lactate transporter, MCT4. Cell nuclei were stained with DAPI. Quantification of mean intensity MCT4 fluorescence in *WT* (*n* = 33) and *GLS^KO^* (*n* = 27) SCCs. Statistical significance (****P < 0.0001) was calculated using a two-tailed *t* test. Scale bars, 100 μm. (**D**) RNA-seq data of *WT* (*n* = 2) or *GLS^KO^* (*n* = 5) tumors showing transcription levels of lactate transporters.

Since ^13^C-glutamine and ^13^C-glucose tracing in *GLS^KO^* tumors revealed decreased glutamine and glucose consumption, we examined whether *GLS^KO^* tumors increased consumption of lactate, another abundant nutrient in circulation. ^13^C-lactate tracing revealed a large increase in lactate uptake in *GLS^KO^* tumors (Fig. 4A) and TCA cycle products, suggesting that increased lactate uptake was able to power the TCA cycle to compensate for the loss of glutaminolysis. We looked for evidence of changes in lactate transporter expression in *GLS^KO^* tumors by staining for monocarboxylate transporter 1 (MCT1) and monocarboxylate transporter 4 (MCT4). These two transporters are known to allow both lactate and pyruvate to traverse the plasma membrane in both directions as needed (*28*). We found that MCT1 expression was unchanged in *GLS^KO^* tumors, but MCT4 was up-regulated in *GLS^KO^* tumors, providing an explanation for the increased lactate uptake and utilization in *GLS^KO^* tumors (Fig. 4, B and C). However, neither MCT1 nor MCT4 was differentially expressed at the RNA level, consistent with a posttranscriptional mechanism by which MCT4 protein is changed in these tumors (Fig. 4D).

**Fig. 5.**
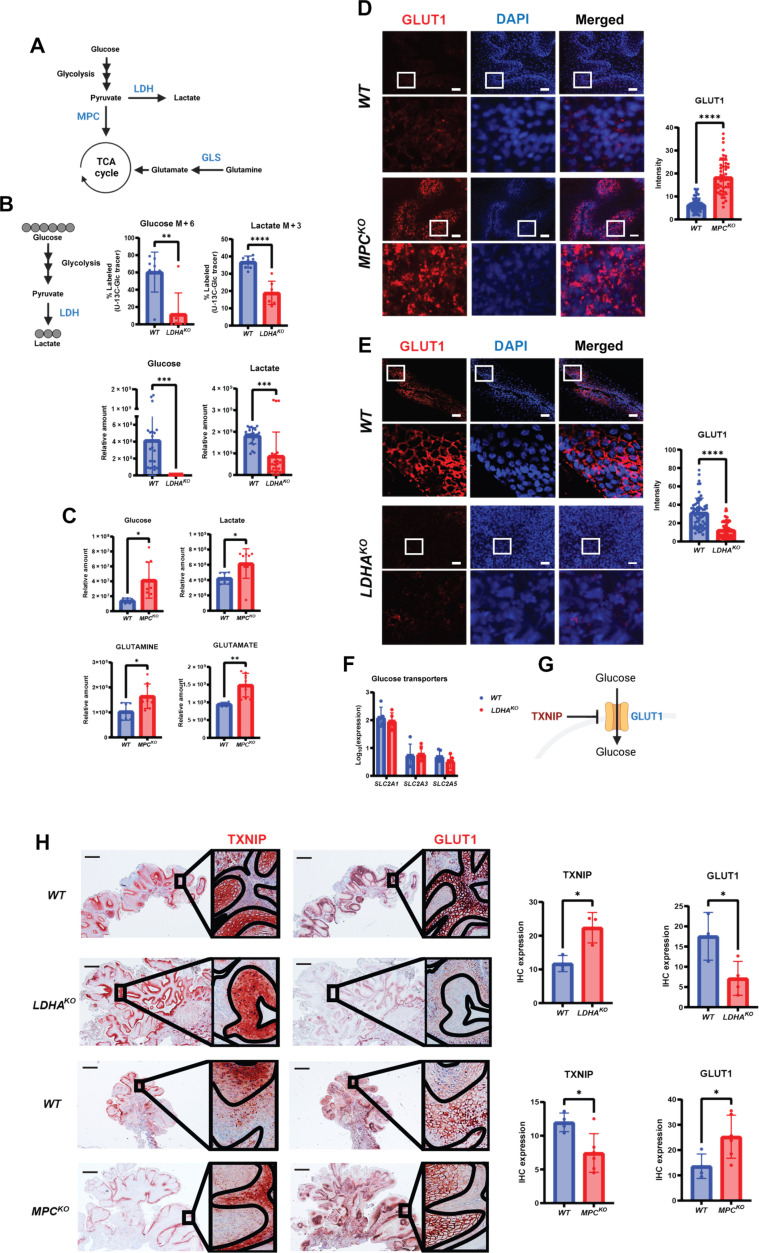
Posttranscriptional increased glucose transporter at the cell surface. (**A**) Schematic of glycolysis, lactate production, and glutaminolysis. (**B**) Schematic of fully labeled glucose isotopomer conversion. Data represent percent of M6-labeled glucose and M3-labeled lactate in tumors [*n* = 8 (*WT*), *n* = 7 (*LDHA^KO^*)] after ^13^C_6_-glucose infusion. Metabolic pool data representing relative amounts of glucose and lactate in tumors [*n* = 24 (*WT*), *n* = 21 (*LDHA^KO^*)]. (**C**) Metabolic pool data representing relative amounts of glucose, lactate, glutamine, and glutamate in tumors [*n* = 6 (*WT*), *n* = 9 (*MPC^KO^*)]. (**D**) *WT* or *MPC^KO^* SCC immunostaining for glucose transporter, GLUT1. Cell nuclei were stained with DAPI. Quantification of mean intensity GLUT1 fluorescence in *WT* (*n* = 56) and *MPC^KO^* (*n* = 49) SCCs. Scale bars, 100 μm. (**E**) *WT* or *LDHA^KO^* SCC immunostaining for glucose transporter, GLUT1. Cell nuclei were stained with DAPI. Quantification of mean intensity GLUT1 fluorescence in *WT* (*n* = 61) and *LDHA^KO^* (*n* = 65) SCCs. Scale bars, 100 μm. (**F**) RNA-seq data of *WT* (*n* = 5) or *LDHA^KO^* (*n* = 5) tumors showing transcription levels of glucose transporters. (**G**) Schematic of TXNIP inhibiting GLUT1. (**H**) *WT*, *LDHA^KO^*, or *MPC^KO^* tumor serial sections were probed for TXNIP and GLUT1. Lines in zoomed images mark boundaries of tissue structures and highlight anticorrelation between TXNIP and GLUT1. Quantification of TXNIP expression [*n* = 3 (*WT*), *n* = 3 (*LDHA^KO^*)] and [*n* = 4 (*WT*), *n* = 6 (*MPC^KO^*)] SCCs. Quantification of GLUT1 expression [*n* = 3 (*WT*), *n* = 4 (*LDHA^KO^*)] and [*n* = 4 (*WT*), *n* = 6 (*MPC^KO^*)] SCCs. Scale bars, 50 μm. Figure 5 (A, B, and G) was produced using BioRender. Statistical significance (*P < 0.05, **P < 0.01, ***P < 0.001, and ****P < 0.0001) for (B) to (E) and (H) was calculated using a two-tailed *t* test.

These results suggesting metabolic flexibility in *GLS^KO^* tumors and capability to switch to a different carbon source through altering expression of nutrient transporters, in particular MCT4, prompted us to reexamine metabolic flexibility and nutrient transporter expression in tumors initiated by HFSCs lacking LDHA. As described previously, *LDHA^KO^* tumors appeared pathologically identical to tumors expressing LDHA (Fig. 1E). We pulsed mice bearing *LDHA^KO^* tumors with ^13^C-labeled glucose before tumor harvesting and, as expected, found diminished tumor glucose uptake, conversion of glucose to lactate, and decreased tumor levels of glucose and lactate, confirming abrogation of glucose metabolism in the absence of LDHA activity described in our previous study (Fig. 5, A and B). In addition, we reexamined metabolomic data from tumors generated by HFSCs lacking the mitochondrial pyruvate carrier (MPC). In this model, tumorigenesis was also unaffected by blocking pyruvate oxidation, providing yet another example of metabolic flexibility (*13*). Increased glucose, lactate, glutamine, and glutamate levels in MPC-null tumors suggested a potential up-regulation of glycolysis and glutaminolysis (Fig. 5C). These data, coupled with our observations about lactate transporter, MCT4, prompted us to ask whether glucose transporters are potentially dynamically regulated to mediate metabolic flexibility. We therefore immunostained for GLUT1, the glucose transporter, and found that GLUT1 protein expression at the cell membrane was strongly down-regulated in *LDHA^KO^* tumors but up-regulated in *MPC^KO^* tumors (Fig. 5, D and E).

Again, RNA-seq showed no changes in the expression of glucose transporter in LDHA-null tumors (Fig. 5F) suggesting a posttranscriptional mechanism for the changes in GLUT1 protein levels. To identify such a mechanism, we looked at expression of thioredoxin-interacting protein (TXNIP), which is known to regulate GLUT1 levels at the plasma membrane (Fig. 5G) (*29*). We stained for these two proteins in the various tumor models described here to see whether a correlation of expression of these proteins could serve to explain the membrane up- or down-regulation of metabolite transporters in response to genetic manipulation of LDHA or MPC. Immunohistochemistry (IHC) for TXNIP and GLUT1 showed a remarkable anticorrelation as previously described, and quantification of total expression for both of these proteins showed that TXNIP is expressed much lower in MPC-null tumors, and the converse was true in LDHA-null tumors (Fig. 5H).

**Fig. 6.**
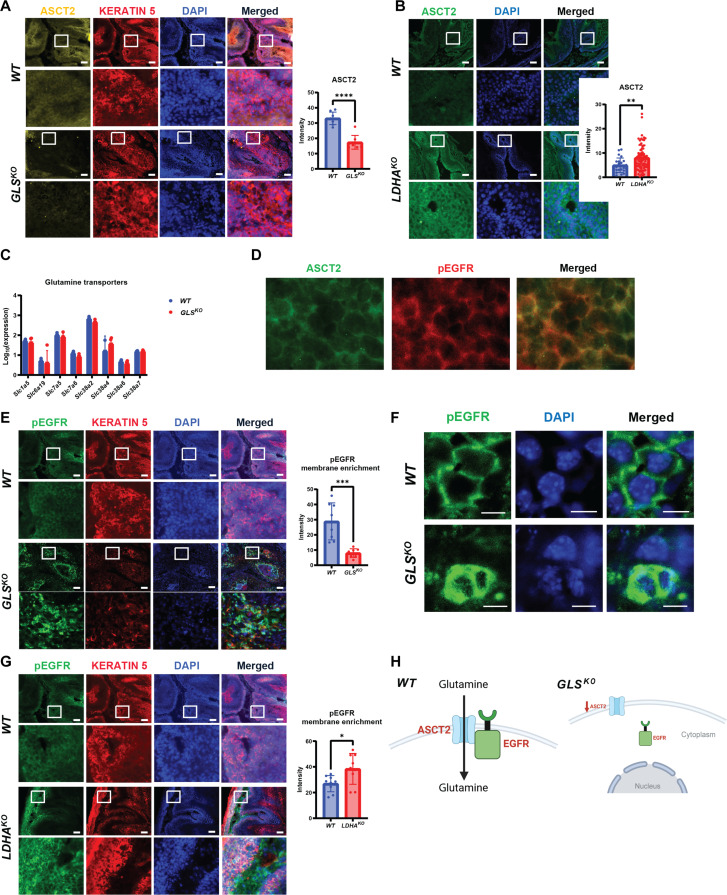
Inhibition of the Warburg effect drives increase in glutamine transporter and uptake. (**A**) *WT* or *GLS^KO^* SCC immunostaining for glutamine transporter, ASCT2. Cell nuclei were stained with DAPI. Quantification of mean intensity ASCT2 fluorescence in *WT* (*n* = 8) and *GLS^KO^* (*n* = 8) SCCs. Statistical significance (****P < 0.0001) was calculated using a two-tailed *t* test. Scale bars, 100 μm. (**B**) *WT* or *LDHA^KO^* SCC immunostaining for glutamine transporter, ASCT2. Cell nuclei were stained with DAPI. Quantification of mean intensity ASCT2 fluorescence in *WT* (*n* = 23) and *LDHA^KO^* (*n* = 74) SCCs. Statistical significance (**P < 0.01) was calculated using a two-tailed *t* test. Scale bars, 100 μm. (**C**) RNA-seq data of *WT* (*n* = 2) or *GLS^KO^* (*n* = 5) tumors showing transcription levels of glutamine transporters. (**D**) Confocal microscopy for *WT* SCC immunostaining for ASCT2 and pEGFR. Scale bar, 20 μm. (**E**) *WT* or *GLS^KO^* SCC immunostaining for pEGFR. Cell nuclei were stained with DAPI. Quantification of pEGFR membrane enrichment in *WT* (*n* = 8) and *GLS^KO^* (*n* = 8) SCCs. Statistical significance (***P < 0.001) was calculated using a two-tailed *t* test. Scale bars, 100 μm. (**F**) Confocal microscopy of *WT* or *GLS^KO^* SCC immunostaining for pEGFR. Scale bar, 10 μm. (**G**) *WT* or *LDHA^KO^* SCC immunostaining for pEGFR. Cell nuclei were stained with DAPI. Quantification of pEGFR membrane enrichment in *WT* (*n* = 9) and *LDHA^KO^* (*n* = 9) SCCs. Statistical significance (*P < 0.05) was calculated using a two-tailed *t* test. Scale bars, 100 μm. (**H**) Schematic proposing ASCT2 and EGFR localizations in *WT* and *GLS^KO^* SCCs. Figure 6H was produced using BioRender.

We next immunostained for ASCT2, a plasma membrane glutamine transporter, in our models of tumors initiated by HFSCs. *GLS^KO^* tumors showed a significant decrease in ASCT2 protein consistent with the decrease in glutamine uptake and metabolism in these tumors (Fig. 6A). ASCT2 protein at the membrane showed a strong increase in LDHA-null tumors, providing a potential mechanism by which glutaminolysis was induced in these tumors (Fig. 6B). Profiling multiple *WT* and *GLS^KO^* tumors using RNA-seq analysis showed that neither ASCT2 nor other relevant transporters were differentially expressed (Fig. 6C). ASCT2 is thought to form a complex with activated epidermal growth factor receptor (EGFR) (*30*), which is also known to be highly active in Ras-driven SCC (*31**,* *32*). We immunostained for active phosphorylated EGFR (pEGFR) and indeed found strong expression at the cell membrane in SCC driven by DMBA/TPA (Fig. 6D), consistent with high EGF signaling activity. In tumors generated by *GLS^KO^* HFSCs, pEGFR expression appeared to be localized to the cytoplasm rather than the plasma membrane, as shown by high-resolution confocal microscopy (Fig. 6, E and F). When looking at RNA-seq analysis for several pathways of downstream EGFR signaling such as mitogen-activated protein kinase (MAPK), phosphoinositide 3-kinase-protein kinase B (PI3K-AKT), and phospholipase C gamma (PLCg), there were scattered differentially expressed genes, but none of the pathways examined pointed to a change in signaling overall (fig. S3E). Moreover, to probe for changes in EGFR signaling at the protein level, we also carried out a series of Western blots and immunostains for antibodies that recognize activity of signaling proteins (fig. S3, F and G). We found that there was an elevation of pEGFR expression in the *GLS^KO^* tumors, despite the fact that there was diminished EGFR enrichment at the cell membrane. Downstream signaling of EGFR, however, was not consistent with the elevated EGFR expression seen in *GLS^KO^* tumors. Despite elevation of EGFR activity as measured by this antibody, this did not correlate with increased tumor progression, perhaps because of the localization of the activity within the cell as opposed to just the overall activity. On the other hand, in tumors from the *LDHA^KO^* background, the pEGFR was at the membrane and expressed at a higher level, similar to what was observed for ASCT2 (Fig. 6G). Therefore, the elevated glutaminolysis observed in *LDHA^KO^* tumors and diminished glutamine uptake observed in *GLS^KO^* tumors could be due to dynamic regulation of active EGFR with ASCT2 at the membrane (Fig. 6H).

**Fig. 7.**
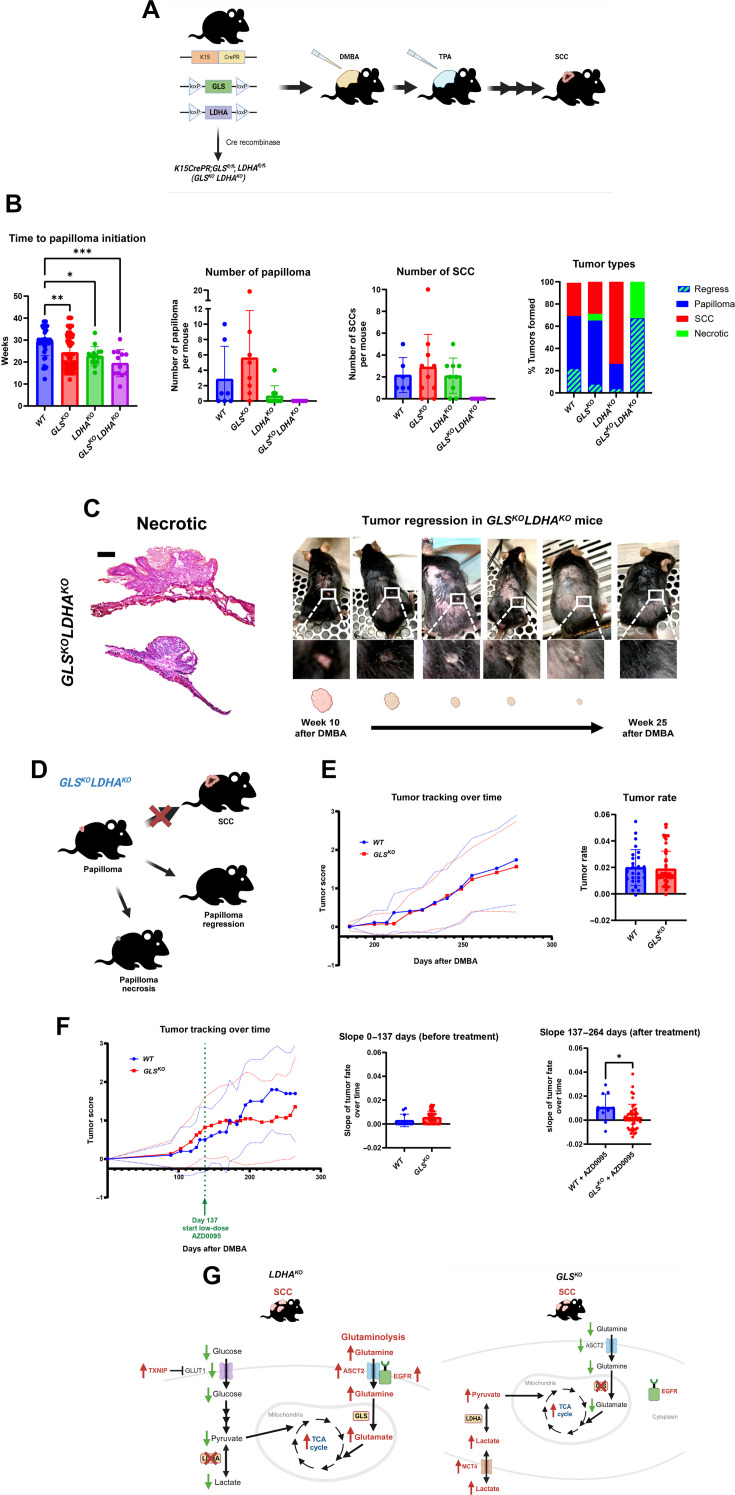
Targeting both glutaminolysis and glycolysis in SCC. (**A**) Schematic of transgenic mice used to knock out GLS and LDHA in HFSCs coupled with topical SCC chemical carcinogenesis using DMBA and TPA. (**B**) Quantification of time to papilloma *n* = 40 (*WT*), *n* = 88 (*GLS^KO^*), *n* = 15 (*LDHA^KO^*), and *n* = 12 (*GLS^KO^LDHA^KO^*) initiation. Each data point represents a tumor of that genotype. Quantification of the number of papilloma [*n* = 6 (*WT*), *n* = 10 (*GLS^KO^*), *n* = 6 (*LDHA^KO^*), *n* = 5 (*GLS^KO^LDHA^KO^*)] and SCC [*n* = 6 (*WT*), *n* = 10 (*GLS^KO^*), *n* = 6 (*LDHA^KO^*), *n* = 5 (*GLS^KO^LDHA^KO^*)]. Each data point represents a mouse of that genotype. Data shown represent tumors present at the end of the experiment. Quantification of percent and types of tumors formed per genotype: *WT* (papilloma = 48%; SCC = 30%; regress = 23%; necrotic = 0%), *GLS^KO^* (papilloma = 57%; SCC = 30%; regress = 8%; necrotic = 6%), *LDHA^KO^* (papilloma = 41%; SCC = 55%; regress = 4%; necrotic = 0%), and *GLS^KO^LDHA^KO^* (papilloma = 0%; SCC = 0%; regress = 67%; necrotic = 33%). (**C**) Necrotic tumors from *GLS^KO^LDHA^KO^* mice stained for H&E. Scale bar, 50 μm. Images of *GLS^KO^LDHA^KO^* mouse over time undergoing papilloma regression. (**D**) Schematic of summary of phenotypic results for *GLS^KO^LDHA^KO^* mice. (**E**) Tumor rates for *WT* (*n* = 26) and *GLS^KO^* (*n* = 73) tumors during DMBA/TPA chemical carcinogenesis. (**F**) Tumor rates for *WT* (*n* = 11) and *GLS^KO^* (*n* = 45) tumors treated with AZD0095 on day 137. Rates quantified before and after AZD0095 treatments. (**G**) Schematic of proposed mechanisms of metabolic flexibility in *LDHA^KO^* and *GLS^KO^* HFSC-induced SCCs. Figure 7 (A, C, D, and G) was produced using BioRender.

The data from Figs. 4 to 6 suggest that SCC-initiating cells have metabolic flexibility for carbon sources to power metabolic pathways, so we hypothesized that perhaps deletion of two carbon sources might be sufficient to starve cells attempting transformation. To test this hypothesis, we crossed animals floxed for both LDHA and GLS with K15-CrePR transgenic mice in an attempt to abrogate both glucose utilization and glutaminolysis (Fig. 7A). We then treated double-floxed mice with DMBA/TPA to induce tumorigenesis. After 10 to 20 weeks, we routinely detected papilloma in all genotypes (Fig. 7B) but never observed the formation of an SCC in *GLS^KO^LDHA^KO^* mice. By the end of the experiment, when the control animals had to be euthanized, there were no papilloma or SCC that lacked both LDHA activity and GLS expression in any of the *GLS^KO^LDHA^KO^* mice (Fig. 7B). This suggests that those papilloma that lacked both LDHA and GLS that might have formed in *GLS^KO^LDHA^KO^* mice probably underwent regression. Careful chronological examination of tumorigenesis in the *WT*, *GLS^KO^*, *LDHA^KO^*, and *GLS^KO^LDHA^KO^* tumors showed that all tumors formed in *GLS^KO^LDHA^KO^* mice were benign papilloma, which then either underwent necrosis or regression (Fig. 7, B to D).

The fact that genetic abrogation of two metabolic pathways blocked cancer formation and the intriguing pattern of nutrient transporter expression in single pathway deletions led us to hypothesize that perhaps coupling genetic blockade of glutaminolysis with small-molecule inhibition of lactate uptake could diminish tumor progression in the DMBA/TPA model. AZD0095 is an established inhibitor of MCT4, the transporter we showed that was up-regulated in *GLS^KO^* tumors (Fig. 4C). We treated both *WT* and *GLS^KO^* tumor model mice with this inhibitor to test whether blocking the primary means of metabolic flexibility would have an effect on *GLS^KO^* tumors. We tracked tumor formation and progression in *WT* and *GLS^KO^* models before and after treatment with AZD0095 and found that, once lactate uptake was inhibited by AZD0095, the trajectory of tumorigenesis was diminished, specifically in tumors arising from deletion of GLS in cancer cells of origin (Fig. 7, E and F). Therefore, these data point toward the utility of blocking multiple metabolic pathways to treat cancer and demonstrate the importance of nutrient transporter regulation as a key mediator of metabolic flexibility.

## DISCUSSION

Together, these data help define the limits of metabolic flexibility of tumor-initiating cells and potentially point toward therapeutic combinatorial strategies to fight cancer initiation and progression. While previous studies argued that blockade of individual metabolic nodes could be an effective treatment strategy, our data argue otherwise. It is worth speculating that the difference between these outcomes could be due to the cancer models being used, namely, in vitro versus in vivo. Our data are consistent with studies showing that instead multiple metabolic pathways must be targeted to overcome metabolic flexibility of cancer cells, including a previous in vivo study in lung cancer showing that tumors that are insensitive to inhibition of glycolysis or GLS alone, are sensitive to the combination of both glycolysis and GLS inhibition (*33*–*35*).

In vivo, cancer cells potentially have access to a much more sophisticated environment due to the presence of vasculature, circulation, lymphatics, the nervous system, the immune system, etc. Therefore, it is possible that blockade of an individual pathway in vitro blocks cell growth simply because nutrients that allow for flexibility are not present in cell culture systems. As a result, therapeutic abrogation of cancer progression will probably require pharmacological targeting of more than one pathway as our genetic data presented here suggest. Fortunately, extensive effort has been devoted to creating small-molecule inhibitors of various metabolite uptake and utilization pathways such as CB839 (GLS), GSK2837808A (LDHA), UK5099 (MPC), and AZD0095 (MCT4). All these compounds show good safety profiles; however, none of these compounds by themselves effectively block tumor progression but perhaps could be more efficacious in combination. It is worth noting that another approach with the drug 6-Diazo-5-oxo-l-norleucine (DON) is beginning to show promise in clinical trials (*15**,* *36**,* *37*). However, this compound appears to target many proteins in the glutamine utilization pathway and perhaps has targets outside of the glutamine pathway, in which case would be consistent with the hypothesis that it is necessary to target multiple metabolic nodes to treat cancer.

Our data also show that when LDHA activity is blocked, an increase of glutamine uptake coincides with the up-regulation of the ASCT2 transporter (Fig. 7G). Conversely, when GLS activity was genetically blocked, lactate uptake was increased along with expression of MCT4 transporter at the cell membrane (Fig. 7G). Furthermore, we expanded previous findings to demonstrate that the GLUT1 transporter is key to the promotion and diminution of glycolytic activity observed in LDHA- and MPC-deleted tumors (Fig. 5). The regulation of these transporters did not appear to be at the transcriptional level, as RNA-seq failed to identify changes in RNA expression of any of these transporters. These results suggest that interactions between these transporters and proteins, such as EGFR and TXNIP, mediate the regulation of cell membrane localization of these transporters, which then appear to drive metabolic flexibility (Fig. 7G). We have yet to identify a mechanism for regulation of MCT4 at the membrane which can explain the increase in lactate uptake in the absence of GLS, but this will be an active area of investigation going forward. The mechanisms proposed here demonstrate that cancer cells have a rather elegant means with which to compensate for loss of function of a metabolic pathway by simply putting more transporter at the membrane for an alternate pathway. Another outstanding question from this study is how cells appear to sense a deficiency in nutrient uptake from one pathway to then up-regulate alternative transporter concentration at the membrane. Others have shown that metabolites themselves can act as signaling molecules by directly binding to protein targets to regulate their activity. Perhaps lactate is the best example of this phenomenon, where proteins can actually be covalently modified by lactylation on lysine residues. On the other hand, it is possible that instead altered levels of particular metabolites trigger a distinct signal that then changes the level of EGFR at the membrane to regulate ASCT2 or TXNIP protein levels to regulate the glucose transporter. TXNIP has been shown in different contexts to be regulated at the RNA level by zinc finger protein 36 (ZFP36) and by microRNAs or at the transcriptional level by oncogenes, cytokines, and growth factors, but our initial efforts to demonstrate a similar mechanism did not yield substantial results. Until there are better described pathways for nutrient expression as a regulator of metabolic flexibility, perhaps small-molecule manipulation of these transporters would be the best way to circumvent metabolic flexibility to treat cancer.

Therefore, it is worth speculating that inhibition of nutrient uptake could be an effective strategy to treat cancer. Of course, our data also point toward the need to inhibit multiple pathways to achieve effective cancer therapy and circumvent the metabolic flexibility described here. We hypothesize that in the double-mutant tumors deleted for LDHA and GLS, HFSCs failed to up-regulate alternative pathways to substitute for the loss of glutaminolysis and lactate utilization as compensatory mechanisms and, as a result, failed to fuel the TCA cycle leading to tumor growth. As a test of these hypothesis, we showed that blocking the activity of a nutrient transporter (MCT4) specifically diminished tumorigenesis in *GLS^KO^* tumors and had no effect on tumorigenesis in GLS-expressing cells (Fig. 7, E and F). Therefore, future efforts will be devoted to both understanding the biochemical mechanisms underlying nutrient transporter expression at cancer cell membranes and developing methods to inhibit metabolic flexibility for the treatment of cancer.

## MATERIALS AND METHODS

### Mice

All animal experiments and related procedures were performed and maintained in accordance with protocols set forth and approved by University of California, Los Angeles (UCLA) Animal Resource Committee and the Institutional Animal Care and Use Committee at UCLA in facilities run by the UCLA Department of Laboratory Animal Medicine. Animal strains came from the Jackson Laboratory (K15-CrePR, GLS1 fl/fl, LDHA fl/fl, and MPC1 fl/fl).

### Two-stage tumorigenesis in mouse skin

Tumors were induced on genetically engineered mice using K15-CrePR animals floxed for either GLS or GLS and LDHA by a cutaneous two-stage skin chemical carcinogenesis (*22*). Transgenic animals were shaved and treated with mifepristone (200 μl of 10 mg/ml dissolved in filtered sunflower seed oil) daily for 3 days by intraperitoneal injection to delete GLS or GLS and LDHA. After 1 week of treatment with mifepristone, mice were topically treated with a tumor-initiating agent, DMBA (400 nmol dissolved in acetone). After 1 week of DMBA application, mice were topically treated with a tumor growth–promoting agent, TPA (20 nmol dissolved in 100% ethanol), twice a week 3 to 4 days apart until time of harvest and 25 to 35 weeks after the initial DMBA treatment. Papilloma began to form 10 to 20 weeks post-DMBA treatment, and SCCs began to form 20 to 35 weeks post-DMBA treatment.

### Histology, immunofluorescence, IHC, and immunoblotting

Tumors were harvested from dorsal skin for each indicated genotype and embedded in unfixed optimal cutting temperature (OCT) compound. Tumors in OCT were cut at 10 μm on a Leica 3200 Cryostat for immunostaining and hematoxylin and eosin staining. For immunofluorescence staining, slides were briefly fixed in 10% buffered formalin and washed in phosphate-buffered saline (PBS) twice for 10 min. Slides were blocked with 10% goat serum/0.25% Triton X-100 for 1 hour at room temperature while rotating. Primary antibodies were diluted into blocking buffer, added to samples, and incubated overnight. The next day, slides were washed in PBS/Tween. Secondary antibodies were added at 1:500 dilution and were incubated on slides rotating at room temperature for 1 hour. Slides were then washed in PBS/Tween, mounted with Prolong Gold with 4′,6-diamidino-2-phenylindole (DAPI; Invitrogen), and sealed with clear nail polish. IHC was performed on formalin-fixed paraffin-embedded tissue sections. Slides underwent antigen retrieval with citrate, were incubated in hydrogen peroxide for 30 min at 4°C, blocked with 10% goat serum/0.25% Triton X-100 for 1 hour at room temperature, and incubated with primary antibodies overnight. For detection, we used a secondary horseradish peroxidase–labeled polymer (Dako) and 3-amino-9-ethylcarbazole (AEC) Substrate Chromogen (Vector Laboratories). For Western blot, the total protein concentration was determined using the bicinchoninic acid (BCA) assay kit (Pierce) per the manufacturer’s protocol with a microplate reader. Ten micrograms of protein was loaded per well for each tumor lysate. Gel was run, transferred, and blocked in 3% BSA. Primary antibodies were diluted in 3% BSA and incubated in membrane overnight. Membrane was washed, incubated with goat anti-rabbit immunoglobulin G (H+L) secondary antibody (horseradish peroxidase; 1:20,000), and imaged using the SuperSignal West Pico PLUS Chemiluminescent Substrate. Table 1 lists the primary antibodies used for immunofluorescence (IF), IHC, and Western blot (WB):

**Table 1.** Antibodies used for IF, IHC, and WB.

Antibody	Source	Identifier	Technique
Anti-rabbit KGA/GAC (GLS)	Proteintech	Catalog no. 12855-1-AP	IF
Anti-chicken KERATIN 5	BioLegend	Catalog no. 905901	IF
Anti-rabbit CD45	Abcam	ab10558	IF
Anti-rat CD11b [M1/70]	Abcam	ab197701	IF
Anti-rabbit MCT1	Proteintech	Catalog no. 20139-1-AP	IF
Anti-rabbit MCT4	Proteintech	Catalog no. 22787-1-AP	IF
Anti-rabbit GLUT1 [EPR3915]	Abcam	ab115730	IF and IHC
Anti-rabbit TXNIP	Invitrogen	Catalog no. yf3950376A	IHC
Anti-rabbit ASCT2 (V501)	Cell Signaling	Catalog no. 5345	IF
Anti-rabbit EGFR (pY1068) [EP774Y]	Abcam	ab40815	IF
Anti-rat Ki67	eBioscience	Catalog no. 41-5698-82	IF
Anti-rabbit cleaved caspase 3	Cell Signaling	Catalog no. 9661S	IF
Anti-rabbit p38 MAPK (pT180/pY182) (D3F9)	Cell Signaling	Catalog no. 4511	WB
Anti-rabbit AKT1 (pS473) + AKT2 (pS474) + AKT3 (pS472)	Abcam	ab192623	WB and IF
Anti-rabbit GSK-3β (pS9) (5B3)	Cell Signaling	Catalog no. 9323	WB
Anti-rabbit c-Jun (pS73) (D47G9)	Cell Signaling	Catalog no. 3270	WB
Anti-rabbit MEK1/2 (pS221) (166F8)	Cell Signaling	Catalog no. 2338	WB
Anti-rabbit ERK1 (pT202) + ERK2 (pT185)	Abcam	ab201015	WB and IF
Anti-rabbit STAT3 (pY705)	Abcam	ab76315	WB
Anti-rabbit CREB (pS133) (87G3)	Cell Signaling	Catalog no. 9198	WB
Anti-rabbit β-actin	Abcam	ab8227	WB

### GLS activity assay

Activity of GLS was measured by using a GLS activity fluorometric assay kit (Biovision, K455) according to the manufacturer’s instructions.

### FDG-PET imaging and analysis

Small-animal PET/computed tomography scans were performed and analyzed as we described in (*13*). Standardized uptake value (SUV) was calculated using %ID/g. ID, injected dose. %ID/g = (SUV divided by animal weight in grams) × 100.

### Tracing with ^13^C_6_-d-glucose, ^13^C_5_-l-glutamine, and ^13^C_3_-sodium-l-lactate

Before euthanasia, mice were intraperitoneally infused with ^13^C_6_-d-glucose (Cambridge Isotope Laboratories, PR-31904), ^13^C_5_-l-glutamine (Cambridge Isotope Laboratories, PR-30230), and ^13^C_3_-sodium-l-lactate (Cambridge Isotope Laboratories, PR-31355) label for 10 min. ^13^C_6_-d-glucose was infused at 2 g/kg, ^13^C_5_-l-glutamine at 0.3 mg/g, and ^13^C_3_-sodium-l-lactate at 200 μl of solution. After 10 min of tracing, tissues were dissected within 3 to 5 min for metabolite extraction. Pulse labeling data represent fractional contribution of metabolites.

### Metabolite extraction and LCMS

These experiments were performed as previously described in (*13*). Briefly, <8 mg of fresh tumors was momentarily rinsed in cold 150 mM ammonium acetate (pH 7.3) and then added into 1 ml of a cold solution of 80% methanol with 10 nM trifluoromethanosulfanate. Tumors samples were homogenized with a tissue homogenizer (BeadBug6 model: D1036, 5 cycles, 4000 speed, 30 times) for full homogenization. After removing insoluble material by centrifugation at 17,000*g* at 4°C for 10 min, the supernatant was added into a glass vial, and metabolites were dried down under vacuum or an EZ-2Elite evaporator. Mass spectrometry was performed as previously described in (*13*). Cell pellets were resuspended in radioimmunoprecipitation assay buffer (Pierce) with Halt protease and phosphatase inhibitors (Thermo Fisher Scientific) on ice. After removing insoluble material by centrifugation at 8000*g* at 4°C for 5 min, total protein concentration was determined using the BCA assay kit (Pierce) per the manufacturer’s protocol with a microplate reader.

### Tumor quantification and scoring

Each tumor developed was tracked over time, and fate was determined on the basis of appearance and histology of tumor. Tumors were scored using the following scoring system: 0 = papilloma regression; 1 = papilloma formation; 2 = papilloma grows; 1 = papilloma gets smaller; 3 = papilloma turns into SCC; 4 = SCC grows; 3 = SCC gets smaller. Slope of each tumor score over time was quantified and noted as tumor rate.

### Topical inhibitor treatments

AZD0095 (MedChemExpress) was dissolved in dimethyl sulfoxide, and mice were treated at 10 times the maximal inhibitor concentration to penetrate the in vivo epidermal barrier. Inhibitor working solutions were then mixed into TPA and applied dorsally twice a week.

### Statistics and reproducibility/statistical analysis

All animals used come from a mixed C57BL6/FVB background with no preference in mouse gender for any studies. There was no statistical measure used beforehand to determine sample size. Data were analyzed in Microsoft Excel and GraphPad Prism, and error bars represent SD between two groups performed by a two-tailed *t* test analysis. Statistical significances were considered if *P < 0.05, **P < 0.01, and ***P < 0.001. Sample size and statistical details can be found in the figure legends.
