# Supplementary material for: Defining metabolic flexibility in hair follicle stem cell induced squamous cell carcinoma
Source: Sci Adv. 2024 Sep 20;10(38):eadn2806. doi: 10.1126/sciadv.adn2806 (PMC11414736; doi:10.1126/sciadv.adn2806)
Supplement: Supplementary file 1 — Figs. S1 to S3 [file sciadv.adn2806_sm.pdf]

Science Advances
AAAS

Supplementary Materials for

Defining metabolic flexibility in hair follicle stem cell induced squamous cell carcinoma

Carlos Galvan *et al.*

Corresponding author: William E. Lowry, blowry@ucla.edu

Sci. Adv. **10**, eadn2806 (2024)
DOI: 10.1126/sciadv.adn2806

This PDF file includes:

Figs. S1 to S3

Supplementary Materials

FIGURE S1 Glutamine

SUPPLEMENTAL FIGURE 1

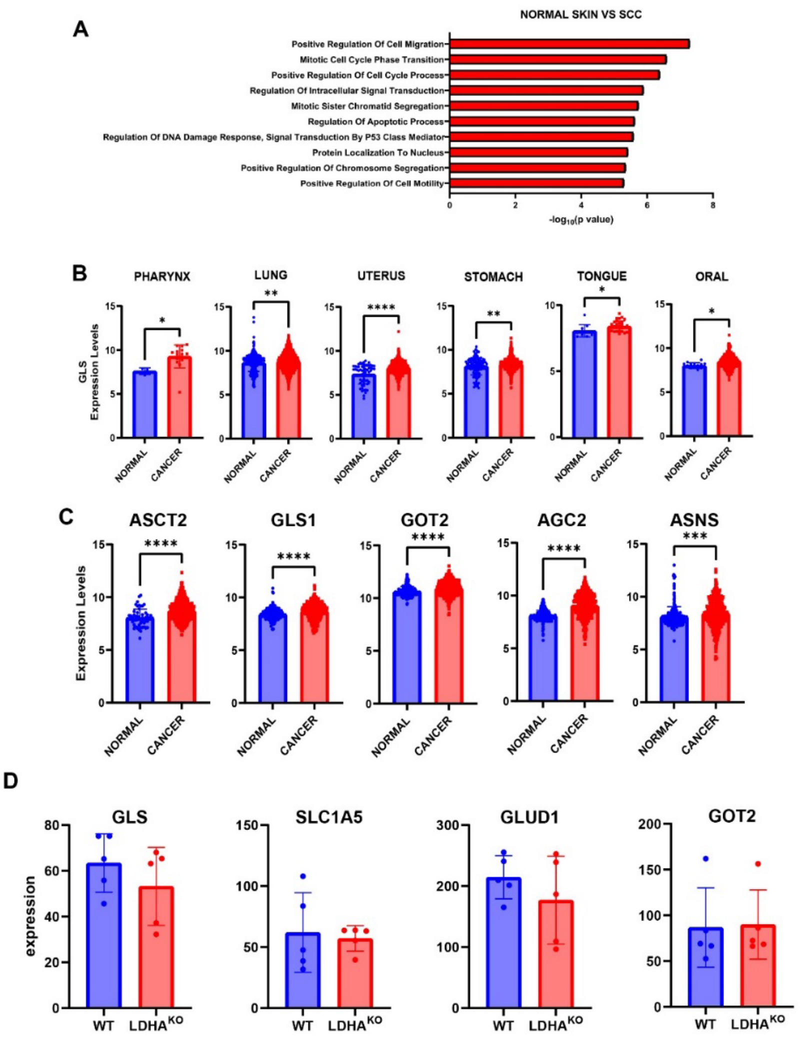

Metabolism is increased broadly in cancers

a) Ontological analysis for biological processes increased in SCC compared to normal skin.
b) GLS gene expression in pharynx n = 3 (normal), n = 15 (cancer); lung n = 508 (normal), n = 2362 (cancer); uterus n = 58 (normal), n = 432 (cancer); stomach n = 117 (normal), n = 1028 (cancer); tongue n = 11 (normal), n = 30 (cancer); oral n = 15 (normal), n = 371 (cancer) based on the Gene Expression database of Normal and Tumor tissue 2 (GENT2) data analysis. Statistical significance (*p<0.05; **p<0.01; ***<0.001; ****p<0.0001) was calculated using a two-tailed t test.
c) ASCT2, GLS, GOT2, AGC2, and ASNS gene expression in skin n = 263 (normal), n = 547 (cancer) based on GENT2 data analysis. Statistical significance (*p<0.05; **p<0.01; ***<0.001; ****p<0.0001) was calculated using a two-tailed t test.
d) RNA-seq data of *WT* (n=5) or *LDHA*^{KO} (n=5) tumors showing transcription levels of genes related to glutaminolysis.

SUPPLEMENTAL FIGURE 2

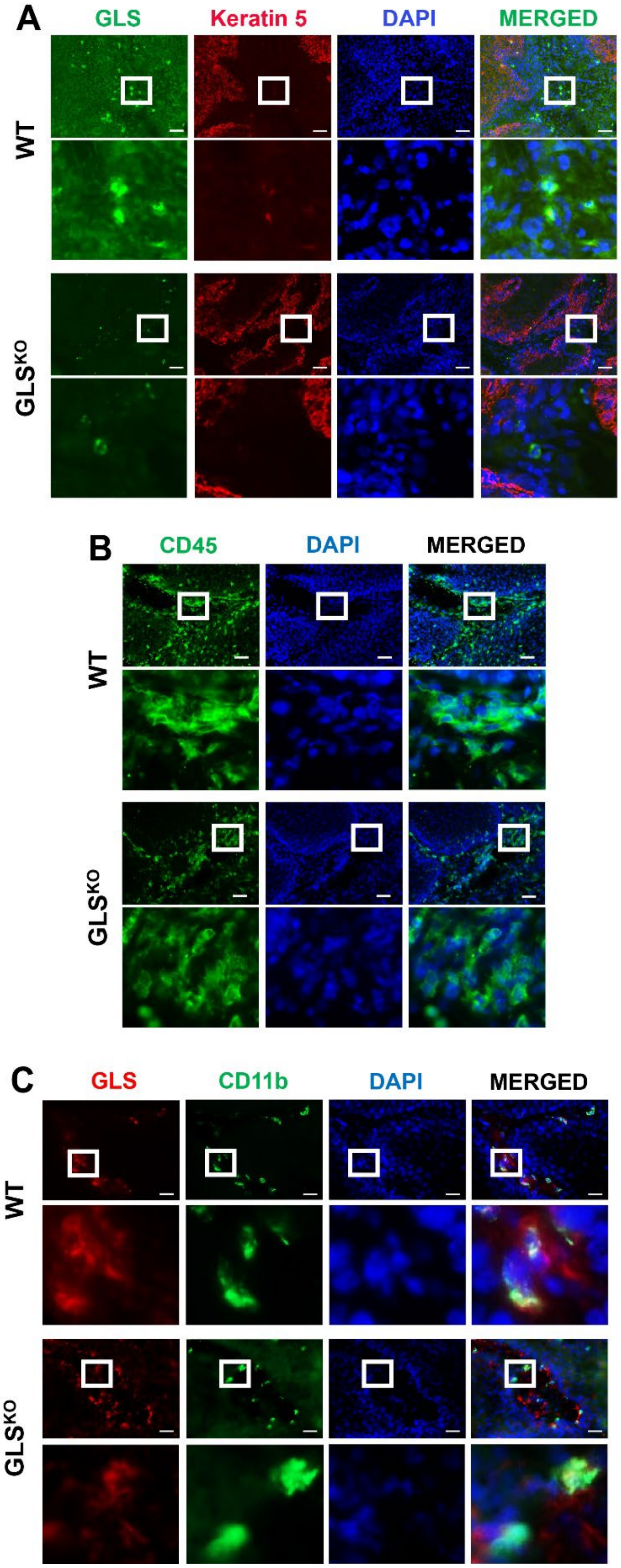

FIGURE S2 GLS+ are present in immune cells in tumor mesenchyme

a) *WT* or *GLS*^{KO} SCC immunostaining for GLS and KERATIN5, an epidermal marker. Magnified images of strong GLS positive cells in mesenchyme of tumor. Scale bar, 100μm.
b) *WT* or *GLS*^{KO} SCC immunostaining for immune cell surface marker CD45. Magnified images of strong CD45 positive cells in mesenchyme of tumor. Cell nuclei were stained with DAPI. Scale bar, 100μm.
c) *WT* or *GLS*^{KO} SCC immunostaining for macrophage marker CD11b. Magnified images of strong CD11b positive cells in mesenchyme of tumor. Cell nuclei were stained with DAPI. Scale bar, 100μm.

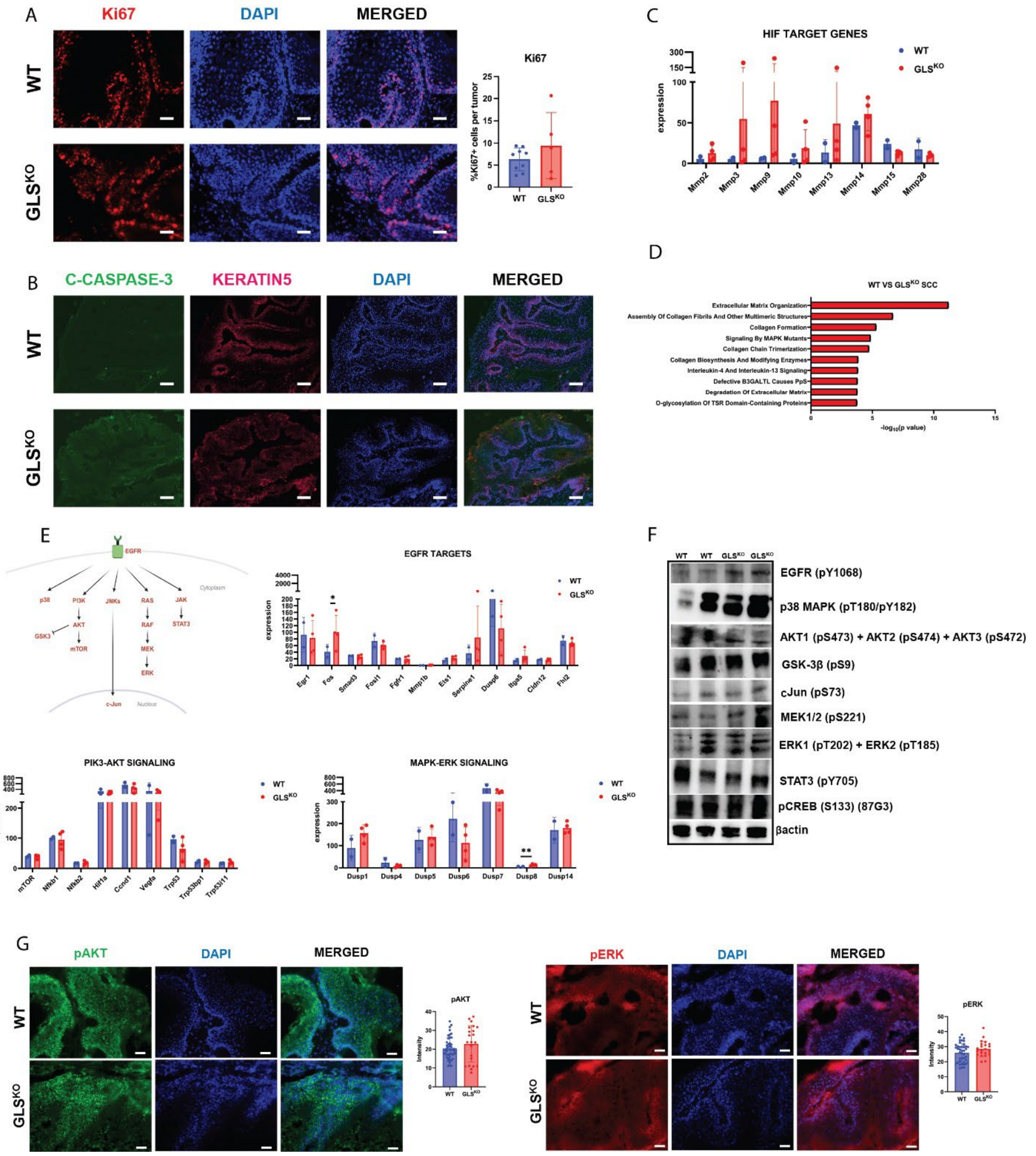

FIGURE S3 Extended characterization of GLS-deleted tumors

a) *WT* or GLS^{KO} SCC immunostaining for Ki67, a proliferation marker, and DAPI for cell nuclei. Quantification of percentage of Ki67 positive cells in *WT* (n=9) and GLS^{KO} (n=5) SCCs. Scale bar, 100µm.

b) *WT* or GLS^{KO} SCC immunostaining for Cleave-Caspase-3, an apoptosis marker, and DAPI for cell nuclei. Quantification of percentage of Cleave-Caspase-3 positive cells in *WT* (n=11) and GLS^{KO} (n=5) SCCs. Scale bar, 100µm.

c) RNA-seq data of *WT* (n=2) or GLS^{KO} (n=5) tumors showing transcription levels of HIF target genes.
d) Ontological analysis for biological processes increased in GLS^{KO} SCCs compared to *WT* SCCs.
e) Schematic of EGFR downstream signaling and RNA-seq data of *WT* (n=2) or GLS^{KO} (n=5) tumors showing transcription levels of EGF target genes: EGFR, PIK3-AKT, MAPK-ERK, and HIF target genes. Statistical significance (*p<0.05; **p<0.01; ***<0.001; ****p<0.0001) was calculated using a two-tailed t test. Figure 3e was produced using Biorender.
f) Western blots of *WT* and GLS^{KO} SCCs probed for proteins associated with activated EGFR signaling.
g) *WT* or GLS^{KO} SCC immunostaining for downstream EGFR signaling, pAKT and pERK. Cell nuclei were stained with DAPI. Quantification of mean fluorescence intensity of pAKT, *WT* (n=46) and GLS^{KO} (n=22), and pERK, *WT* (n=41) and GLS^{KO} (n=22), in SCCs. Scale bar, 100μm.
